# Submersed Aquatic Vegetation Enhances Density and Diversity of Epifaunal Invertebrates Compared to Filamentous Mats in the Central Baltic Sea

**DOI:** 10.1002/ece3.71498

**Published:** 2025-05-29

**Authors:** Chiara D'Agata, Thomas A. B. Staveley, Johan S. Eklöf, Jonathan S. Lefcheck, Gunilla Rosenqvist, Lina Mtwana Nordlund

**Affiliations:** ^1^ Department of Earth Sciences, Natural Resources and Sustainable Development Uppsala University Visby Sweden; ^2^ Department of Aquatic Resources, Institute of Freshwater Research Swedish University of Agricultural Sciences Drottningholm Sweden; ^3^ Department of Ecology, Environment and Plant Sciences Stockholm University Stockholm Sweden; ^4^ University of Maryland Center for Environmental Science Cambridge Maryland USA; ^5^ Department of Earth Sciences, Natural Resources and Sustainable Development, Blue Centre Gotland Uppsala University Visby Sweden

**Keywords:** benthic vegetation, coastal zone, Gotland, macrophytes, SAV, Sweden, vegetation structure

## Abstract

Submersed aquatic vegetation (SAV) provides essential habitat and food to numerous coastal invertebrate species. In the eutrophic Baltic Sea, fast‐growing drifting algae form extensive mats that can negatively impact SAV. However, these mats also offer additional habitat and food to epifauna. The aim of this study was to assess the effects of SAV and filamentous mats on epifaunal communities in shallow soft‐bottom habitats around Gotland, Sweden, in the central Baltic Sea. We used generalised linear models (GLMs) to evaluate the influence of SAV vertical structure, biomass and macrophyte species richness (including macroalgae) and filamentous mat biomass on epifaunal community properties as well as on those of key grazer species. Diversity, vertical structure and biomass of SAV were positively associated with higher total epifaunal abundance and greater abundance gastropod grazers. In contrast, filamentous mats only increased gastropod abundance and biomass. In addition to introducing a rapid tool for quantifying vegetation structural complexity, this study highlights the selective effects of different habitat types on invertebrate communities in a relatively understudied region of the Baltic Sea. As warming temperatures and eutrophication promote filamentous mat growth, reducing nutrient pollution and protecting SAV will be crucial for sustaining abundant and diverse epifaunal communities.

## Graphical abstract

This study evaluates the relative importance of Submersed Aquatic Vegetation (SAV) and filamentous mats in shaping epifaunal invertebrate communities in the central Baltic Sea. Our results showed that diversity, vertical structure, and biomass of SAV were all positively associated with higher total epifaunal abundance and greater abundance of crustacean and gastropod grazers, while filamentous mats only positively influenced gastropods. Our results highlight the importance of managing for diverse, abundant, and complex SAV assemblages.

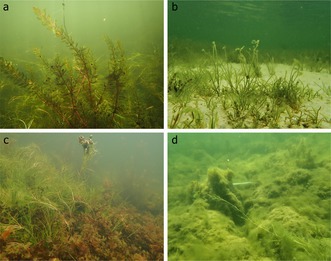

## 1 Introduction

Submersed aquatic vegetation (SAV) such as marine and freshwater angiosperms play a key role in supporting coastal faunal communities. By attenuating water flow, SAV facilitates the settlement of pelagic larvae (Eckman 1987; Newell et al. 2010) and promotes sedimentation of particulates, thus improving water clarity (Orth et al. 2006). SAV tissues are a direct food source for diverse species (Bakker et al. 2016; Lodge 1991; Valentine and Heck 1999). Their structure offers shelter and nursery habitats to numerous fish and invertebrates (Heck et al. 2003; Heck and Thoman 1981; Lefcheck et al. 2019), as well as an attachment surface for epibiota (Kouchi et al. 2006; Thornber et al. 2016). The invertebrate communities associated with coastal SAV comprise species with diverse life histories and functional traits, which include grazing crustaceans such as isopods and amphipods, and gastropods (Lefcheck et al. 2017; Orth et al. 1984). These grazers control fouling epibionts, reducing competition for light and increasing SAV productivity (Duffy and Harvilicz 2001; Neckles et al. 1993). SAV‐associated invertebrates are also key food items for many migrating fish, thus they play an essential role in mediating the transfer of primary productivity to higher trophic levels and linking coastal and offshore habitats (Heck et al. 2008; Orth et al. 1984; Sobocinski and Latour 2015).

The relationships between SAV and invertebrates are complex, and various aspects of SAV have been shown to influence invertebrate communities. For instance, positive relationships were detected between SAV biomass and invertebrate diversity and abundance (Attrill et al. 2000; Heck and Wetstone 1977; Stoner 1980; Virnstein et al. 1984), SAV percent cover and invertebrate diversity and abundance (Reed and Hovel 2006), and SAV surface area and invertebrate diversity (Sirota and Hovel 2006), abundance and biomass (Parker et al. 2001). Studies investigating the responses of invertebrates to specific morphological traits of SAV have also shown that leaf shape (Kenyon et al. 1997), plant morphological complexity (Hansen et al. 2011), and amount of vertical structure (Kouchi et al. 2006) influenced habitat choice and colonization of epifauna. Across heterogeneous seascapes, these properties can significantly influence how the SAV habitat is utilized (Murphy et al. 2021).

Globally, coastal ecosystems have been affected by eutrophic conditions caused by a combination of human activities, including coastal development, nutrient pollution, overfishing and climate change (Malone and Newton 2020). Eutrophic conditions favor the overgrowth of filamentous algae, which have a competitive advantage over large macrophytes in exploiting both nutrients and light (Arroyo and Bonsdorff 2016; Duarte 1995). Other than growing epiphytically and on other hard substrates, filamentous algae can be found as drifting mats. These form when detached branches or free‐living algal species aggregate, transported by winds and water currents (Arroyo and Bonsdorff 2016). Although these habitats can provide provisional additional structure and food resources to faunal communities (Boström and Bonsdorff 2000; Salovius et al. 2005), an increase in mat cover, biomass and length of occurrence can lead to loss of SAV and the structural complexity they provide (Muguerza et al. 2017). Given the importance of the vegetation's structural features in shaping invertebrate communities, this can ultimately lead to a reduction in epifauna biomass and abundance (Lanari et al. 2018). Previous studies have shown substantial differences between epifaunal communities associated with SAV versus drift algae (Knowles and Bell 1998; Lefcheck et al. 2017; Virnstein and Howard 1987) and even among different species of drift algae (Ramus et al. 2022).

The Baltic Sea is a large brackish water body in Northern Europe, connected by a narrow passage to the North Sea. The limited influx of saltwater from the North Sea, together with abundant freshwater runoff, results in a characteristic strong north–south salinity gradient, ranging from nearly freshwater conditions in the north to polyhaline (over 20 PSU) in the south (Snoeijs‐Leijonmalm and Andrén 2017). The gradient affects the distribution of organisms, which include species of both marine and freshwater origins (Kautsky and Kautsky 2000; Leppäranta and Myrberg 2009; Snoeijs‐Leijonmalm and Andrén 2017).

In shallow soft bottom habitats, the benthic vegetation community comprises approximately 40 species of macrophytes (Hansen 2016). These include aquatic plants such as pondweeds (*Potamogeton* spp. and *Stuckenia* spp.) and water milfoils (*Myriophyllum* spp.), which often dominate the assemblages (Austin et al. 2021; Hansen 2008). Seagrasses such as *Zostera marina* are less common, as in the Baltic Sea they are limited by the salinity gradient and displaced by competition from other SAV (Boström et al. 2014). The macroalgae *Fucus vesiculosus* and *Furcellaria lumbricalis* occur in these habitats in their free‐living form (Kotta and Orav 2001; Preston et al. 2024). Overall, these macrophytes support more diverse epifaunal communities compared to bare sand patches (Boström and Bonsdorff 1997; Henseler et al. 2019). Epifauna are mainly represented by mesoinvertebrates, in particular gammaridean amphipods, *Idotea* isopods, gastropods, and insect larvae (Boström and Bonsdorff 1997; Kautsky and Kautsky 2000; Korpinen, Honkanen, et al. 2007; Korpinen, Jormalainen, et al. 2007; Korpinen and Westerbom 2010; Leidenberger et al. 2012).

Drifting mats of filamentous algae (herafter: filamentous mats) in the Baltic Sea typically include chlorophytes in the genera *Chaetomorpha* and *Cladophora*, the phaeophytes *Pilayella* and *Ectocarpus* and the rhodophyta *Ceramium* (Bonsdorff 1992). Additionally, high volumes of filamentous cyanobacteria such as *Aphamizonenon*, *Nodularia* and *Dolichospermum* can be found in these mats (Olofsson et al. 2020). Since 1970s, increased nutrient load has led to the proliferation of filamentous species across the Baltic Sea (Olofsson et al. 2020; Pihl et al. 1995). Coastal areas that are highly impacted by eutrophication are particularly vulnerable to the overgrowth of filamentous mats, and these can be found covering large coastal areas (Berglund et al. 2003; Bonsdorff et al. 2002).

Located centrally in the Baltic Proper basin, the island of Gotland, Sweden, provides a valuable case study for coastal epifaunal communities. With few exceptions (e.g., Ljungberg 2015), the role of benthic structures in supporting epifaunal communities in this region remains understudied, and little is known about the relationships between filamentous mats, SAV and epifaunal communities from naturally occurring communities.

The objective of this study was to address this knowledge gap by providing new insights into the ecology of shallow water habitats in this region of the central Baltic Sea. We expected that: (i) overall epifauna abundance and diversity would respond positively to SAV characteristics that increase habitat availability and structural complexity, such as biomass and vertical structure; (ii) that, similarly, epifauna abundance and diversity would respond positively to overall macrophyte diversity (SAV and habitat‐forming macroalgae); and (iii) grazers would positively respond to the additional habitat and food source provided by filamentous mats. To capture the role of habitat complexity, we also developed a new metric for vertical structure. We used generalised models (GLMs) to test these effects, including also two environmental variables known to strongly influence epifaunal communities, depth and fetch (Boström et al. 2006; Korpinen and Westerbom 2010; Råberg and Kautsky 2007; Wallin et al. 2011) as well as location (bay), to account for eventual local differences.

## 2 Materials and Methods

### 2.1 Field Surveys

The island of Gotland is situated in the Baltic Proper, the largest subsystem in the Baltic Sea, with salinity ranging between 5.0 and 7.5 PSU. In comparison with other islands in the Baltic Sea, Gotland is geographically distant from continental coastlines and other major islands, including the topographically complex Åland islands and Archipelago Sea in the north. Gotland's coastline provides diverse marine habitats, such as large shallow inlets and bays, many of which are of high ecological value and of European importance (Ruskule et al. 2023).

#### 2.1.1 Station Selection and Set‐Up

**FIGURE 1:**
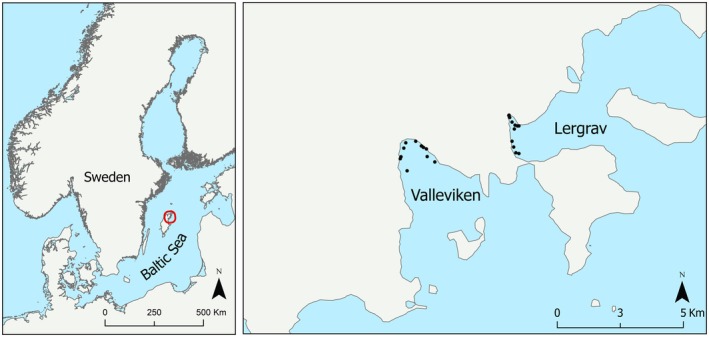
Map of the study stations on the north‐west coast of Gotland, Sweden.

Field surveys were conducted in two bays, Valleviken and Lergrav, on the north‐east coast of Gotland, Sweden, during 4 weeks in July and August 2021 (Figure 1). The bays were chosen as they had extensive shallow‐water soft bottom substrates, had relatively low boat traffic, and had similar exposure to the prevailing south‐westerly winds. Sampling was conducted during July–August as it coincides with the period of highest productivity in the Baltic Sea, which includes the peak in drifting algal biomass in this region (Berglund et al. 2003).

**FIGURE A1:**
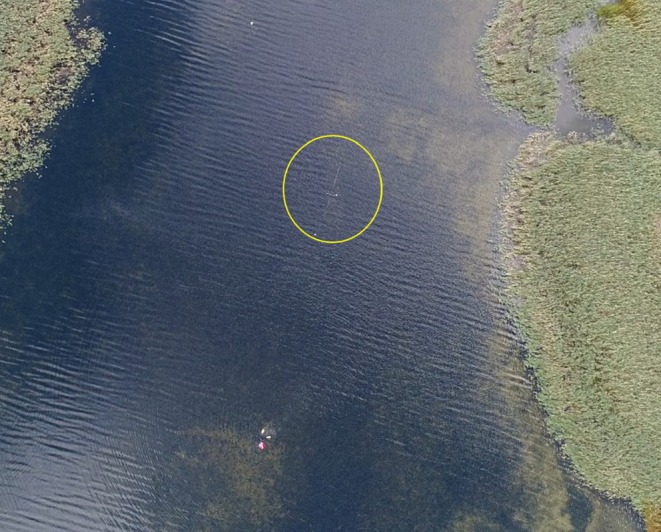
Aerial view of the estimated area (here highlighted by the yellow circle) of a sampling station. Visible, in the middle, a weighted buoy and a measuring tape to delineate a 10 m diameter. Photo: Daniel Olsson.

Vegetation and faunal communities were surveyed at 21 sampling stations: 10 were located in Valleviken and 11 in Lergrav. Areas with water depths between 0.5 and 30 m were identified with the aid of nautical maps. The stations were placed randomly within these areas, provided that there was a minimum distance of at least 30 m between stations and a vegetation cover within them of at least 5% (rapidly assessed by snorkelling). Each station consisted of an estimated circular area marked with a weighted buoy at the center and an attached measuring tape to delineate a 10 m diameter (station area: 78.54 m^2^, Figure A1).

#### 2.1.2 Environmental Variables

Depth (m) and water temperature (°C) were measured at the center of each station using a diving computer (SUUNTO Zoop Novo). Depth measurements were adjusted with respect to mean sea level. The average fetch per station (m) was calculated with the *windfetch* package (Seers 2021) in the R environment v. 4.3.1 (R Core Team 2023), using the GPS position at the surface of the middle of the station and the default setting for maximum distance of 300 km. In addition, three water samples per station were collected and stored for subsequent analysis of salinity (PSU) with a multimeter (Elma 795).

#### 2.1.3 Area‐Based Variables: Estimations of Vegetation Cover, Vertical Structure and Composition

The percent cover of each macrophyte species (SAV and large, non‐filamentous macroalgae) was estimated within three 0.5 × 0.5 m (0.25 m^2^) square quadrats randomly placed within each station. The cover was intended as the two‐dimensional view of the overall vegetation from above. The percentages were recorded in 5% increments, allowing for the value of 1% for single occurrences. When the identification of a macrophyte species could not be carried out in situ, a specimen was collected for later identification in the laboratory. Average values were calculated from the three quadrats at each station.

In the same quadrats, vegetation vertical structure was characterised by estimating the % cover (LC) of vegetation height across five discrete layers: less than 10, 10–30, 30–50, 50–70 and over 70 cm (modified from Schulz et al. 2009, maximum cover = 100%). To each height layer, we assigned a weighted value from 1 to 5, with ‘1’ corresponding to the lowest vegetation height layer (less than 10 cm) and ‘5’ to the tallest layer (over 70 cm). This allowed the volume of vegetation in different layers (LV) to contribute differently to the overall index, depending on how tall the vegetation was. Finally, a standardised vertical structure index (VI, hereafter referred to as the vertical index) was calculated for each station using the following formula:

$$\mathrm{VI}=\frac{\sum_{i=1}^{5}{\mathrm{LC}}_{i}\cdot LV_{i}}{500}$$

where, for each station, LC_*i*_ (layer vegetation cover) is the % cover of vegetation in the *i*th height layer and LV_*i*_ (layer vertical contribution) is the weighted value assigned to the *i*th height layer (ranging 1–5). The sum of LC_*i*_ across all layers equals 100%, representing the vertical distribution of the total vegetation cover. The denominator (500) reflects the maximum possible value, where 100% vegetation cover occurring in the tallest layer (over 70 cm, weighted value = 5) would result in 500.

For the analysis, we used the average values from the three quadrats at each station. This method required minimal time in the field since the % of vegetation at any given height layer was visually estimated, without the need for direct measuring or destructive sampling.

#### 2.1.4 Macrophyte Species Richness

Within each station, visual inspection of the entire area was conducted to detect less common angiosperm or large non‐filamentous macroalgae species that were not recorded within the quadrats. The macrophyte species richness was subsequently calculated by merging these additional species with those recorded in the quadrats and sampling bag for each station (see following section on quantitative sampling). The only taxa excluded from this calculation were those forming the drifting filamentous mat.

#### 2.1.5 Quantitative Sampling: Macrophytes, Filamentous Mats and Macroinvertebrates

A quantitative sample of macrophytes, together with associated filamentous mats and epifaunal invertebrates (≥ 1 mm), was collected at a randomly chosen location within each station. A mesh bag with a rigid circular opening (radius: 0.18 m, area: ~0.1 m^2^, mesh size < 0.5 mm, organisms < 1 mm were not considered for this study) was used to collect the samples. The bag was carefully lowered over the vegetation and, once fully encompassed, the vegetation was cut just above the substrate and brought to the surface. The samples were then placed on ice in a cooler box until final storage at a temperature of −20°C. In the laboratory, macrophytes, drifting filamentous mats and macroinvertebrates were then separated. Macrophytes and, if present, epiphytes were sorted and identified to species or genus level.

The filamentous mats were composed of tightly intertwined filamentous algae and cyanobacteria. They were not sorted into individual species, but subsamples were taken at random to identify the species composition of the algal fraction of the mat. To this end, small portions of the mats were examined under a microscope (Motic SMZ‐171‐TP) and the filamentous algae were identified to species or genus level. Cyanobacteria were not further identified. In the analysis, all the species composing the drifting mats were collectively pooled as the ‘filamentous mat’, but the higher taxonomic resolution for the filamentous algae allows for a better characterisation of the overall community (see Table 2).

The invertebrates were sorted, identified to the species level where possible, and counted. The bryozoan *Einhornia crustulenta* was only found in two samples, where it formed small colonies (< 1.5 cm across). As the exact abundance of these colonial organisms can be challenging to measure (Hansen et al. 2011), one colony was noted as the equivalent of one recorded individual. The same approach was used for the uncommon hydrozoan *Cordylophora* spp.

For the analyses of the focus grazers, four groups were identified: (1) *Gammarus* amphipods, (2) *Idotea* isopods, (3) gastropods of the family Hydrobiidae and the invasive *Potamopyrgus antipodarium* (also until recently collocated within the Hydrobiidae family; Butkus et al. 2020), hereafter referred jointly to as ‘Hydrobiidae’ for simplicity, and (4) the gastropod *T. fluviatilis* . This grouping allowed for the inclusion of the most important grazers in the system (Goecker and Kåll 2003), whereas also preserving key differences in their morphological traits. These were specifically: (a) feeding apparatus: jaw in *Gammarus* and *Idotea* and radula in the gastropods; (b) mobility: swimming, rafting and crawling in *Gammarus* and *Idotea*, rafting and crawling in the gastropods; (c) type of protection: soft exoskeleton in *Gammarus* and *Idotea*, hard shell in the gastropods and (d) body shape: laterally compressed in *Gammarus*, elongated in *Idotea*, conical in the Hydrobiidae and semiglobose in *T. fluviatilis* (a–c based on Törnroos and Bonsdorff 2012).

The dry weight per sample (expressed as grams per bottom area sampled of 0.1 m^2^) of each macrophyte, epifaunal species and of the filamentous mat were taken after drying at 60°C for 14 days. A list of all epifaunal and vegetation taxa found in these samples are reported in Table 2.

### 2.2 Statistical Analysis

All statistical analyses and plotting were conducted in the R environment v. 4.3.1 (R Core Team 2023).

We explored the effects of environmental and vegetation variables on the epifauna community attributes by applying generalized linear models (GLMs). Depending on the likelihood distribution, we used the *stats*, *MASS* v. 7.3‐61 (Venables and Ripley 2002) or *glmmTMB* v. 1.1.10 (Brooks et al. 2017) packages.

The models' initial suite of predictors included eight vegetation measures. Of these, four were derived from the cover‐based estimations (i.e., percent cover of SAV, percent cover of *F. vesiculosus* , percent cover of the filamentous mat and vertical index), three from the quantitative sampling (i.e., SAV dry biomass, *F. vesiculosus* dry biomass and filamentous mats dry biomass), and one representing macrophyte richness. The macroalga *F. vesiculosus* was initially added to the analysis as it is an important habitat‐forming species in the Baltic Sea, also in soft bottoms (Austin et al. 2021). However, its inclusion resulted in the occurrence of multiple model convergence issues. As this macroalga is not central to our research question, *F. vesiculosus* was excluded from further analysis. To the models, we added two key environmental variables, depth and fetch, as well as ‘Bay’ as a fixed factor with two levels.

The predictor variables were then checked for multicollinearity by calculating the variance inflation factor (VIF) using the *usdm* package *v*.2.1‐7 (Naimi et al. 2014). Variables with a high level of collinearity (VIF‐values ≥ 5) were then removed from the GLMs (Zuur et al. 2010). Vegetation % cover and filamentous mat % cover were removed, as they were highly correlated with, respectively, vertical index (*r* = 0.76, *p* < 0.001) and filamentous mat biomass (*r* = 0.85, *p* < 0.001). The final set of variables included two environmental predictors (fetch and depth) and four vegetation community predictors (vertical index, macrophyte richness, SAV biomass and filamentous mat biomass). A list of the final suite of predictors used, along with the rationale for their inclusion, is provided in Table 1.

**TABLE 1:** Background to the choice of predictors in the study, the metric used in the GLMs, and notes on how they were measured.

Premise	Predictor	Metric	Measurement
The distribution of organisms is influenced by depth (Korpinen and Westerbom 2010; Råberg and Kautsky 2007)	Depth	Log_10_ (m)	One measurement taken at the middle of the station
The distribution of epifauna is influenced by exposure level (Boström et al., 2006; Korpinen and Westerbom 2010; Råberg and Kautsky 2007; Wallin et al. 2011)	Fetch (proxy for exposure)	Square root (m)	Average per station calculated on 10° increments based on GPS coordinates
Epifauna diversity and abundance are influenced by aboveground plant biomass (Aleixo et al. 2022; Heck and Wetstone 1977; Stoner 1980; Stoner and Lewis 1985)	SAV biomass	gDW	Dry biomass per sample (0.1 m^2^)
Faunal communities are influenced by vegetation volume and vertical structure (Cunha et al. 2012; Hirst 2007)	Vertical index	Index score	Index based on % cover of discrete height layers (average of three 0.25 m^2^ quadrats)
Epifaunal communities are positively influenced by macrophyte species richness (Ramus et al. 2022)	Macrophyte richness	Total number of species	Cumulative number of species recorded in both qualitative and qualitative samples at each station
Filamentous algae provide additional habitat and food to epifauna (Korpinen et al. 2008; Salovius et al. 2005)	Filamentous mat biomass	Square root (gDW)	Dry biomass per sample (0.1 m^2^)
Bay	Valleviken/Lergrav		Location of sampling station

The influence of the predictors was tested on total epifauna abundance as well as on several measures of diversity: species richness, Shannon diversity and Pielou's evenness. Species richness reflects the total number of epifauna species in a sample. Shannon diversity, which accounts for species' relative abundances, was converted to effective (Hill) numbers by exponentiation (Roswell et al. 2021). Pielou's evenness measures how evenly individuals are distributed among species (Pielou 1966). The same suite of predictors was used to analyze the abundance and biomass of the four grazer groups: (a) *Gammarus*, (b) *Idotea*, (c) Hydrobiidae and (d) *T. fluviatilis*. To resolve convergence issues in two models (*Gammarus* abundance and *Gammarus* biomass), we fitted the models using the *glmmTMB* package with the BFGS optimization method.

We modelled Hill's numbers and Pielou's evenness of epifauna using a Gaussian (log‐link) and a Beta (logit‐link) distribution, respectively. Total epifaunal abundance, species richness and the abundance of *Gammarus*, *Idotea*, Hydrobiidae and *T. fluviatilis* were modelled using a negative binomial distribution with a log‐link. Biomass of these same grazer groups was analysed using a Gamma distribution with a log‐link.

Diagnostic QQ‐plots produced with the *DHARMa* package *v*. 0.4.7 (Hartig 2022) were used to evaluate residual distributions. Because we were testing the same question using multiple indicators derived from the same community (survey), we applied a Bonferroni correction to account for the increase in Type I error associated with multiple testing, recognising that this test is conservative and may increase the risk of Type II error. The traditional threshold of *α* = 0.05 was therefore adjusted to 0.007 to denote statistical significance. Finally, standardised effect sizes were calculated using the packages *piecewiseSEM v*.2.3.0.1 (Lefcheck 2016) or *performance v*.0.12.4 (Lüdecke et al. 2021). Standardised effect sizes are expressed in terms of standard deviations of the mean, which allows for meaningful comparison across variables measured in different units. This approach enables the assessment of the relative contributions of predictors with varying units, such as biomass, species richness, Pielou's evenness and vertical index. Both standardised and unstandardised (raw units) are reported. To visualise the relationships between significant predictors and response variables, partial regression plots were generated using the package *effects v*.4.2‐2. (Fox and Weisberg 2018).

## 3 Results

### 3.1 General Description of the Environmental Characteristics and Vegetation Community

Salinity and temperature showed low variability between stations (salinity: mean = 6.6 ± 0.3 PSU [SD]; temperature: mean = 20.1°C ± 1.8°C [SD]). The average wind speed during the sampling period was 5.7 ms^−1^ (range = 0–13 ms^−1^), with prevailing direction SW, which is typical for this area at this time of year (SMHI 2023).

**FIGURE 2:**
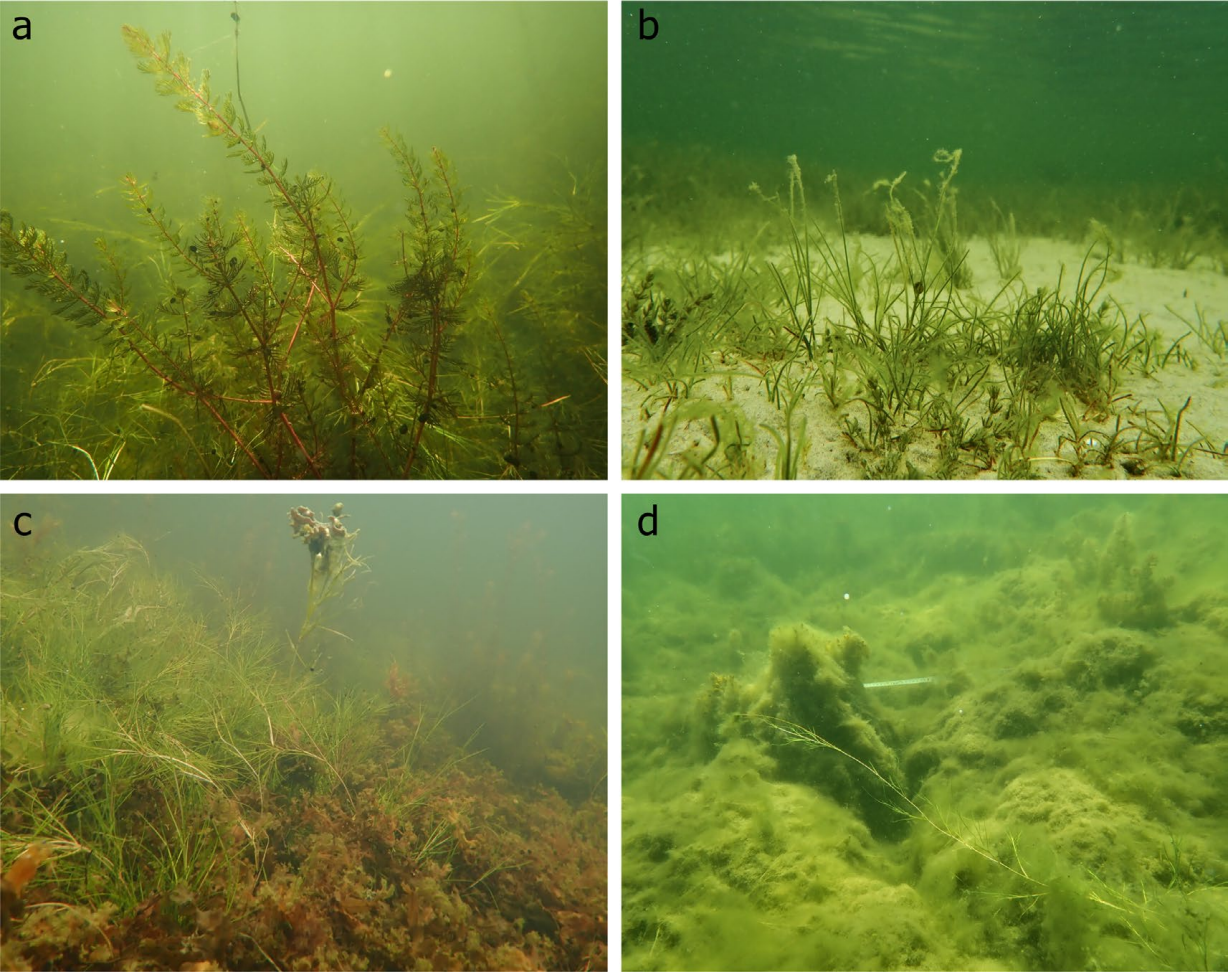
Vegetation assemblages in the sampling stations. Habitats (a) dominated by tall (> 70 cm) SAV: *Myriophyllum spicatum* (foreground) and *Stuckenia pectinata* (background); (b) dominated by short (< 10 cm) SAV: *S. pectinata* ; (c) mixed macrophytes with *S. pectinata* and free‐living *Fucucs vesiculosus* and (d) filamentous mats covering benthic vegetation. Photo: Chiara D'Agata.

Across all the stations, the overall macrophyte community was composed of 18 taxa, consisting of five angiosperms, three benthic macroalgae and ten filamentous species, most of which were found exclusively as part of the filamentous mats (Table 2). The macrophyte species richness per sample was generally low, with a maximum of four species and a mean of 2.57 ± 0.98 (SD) species (Table A1). Among the macrophytes, SAV was the dominating group, accounting for 88% of the cover and 83% of the dry biomass. The most abundant species were *Stuckenia pectinata* and *Myriophyllum spicatum* (Table A1, Figure 2a,b). Among the benthic macroalgae, the dominant species was *F. vesiculosus* , primarily encountered in its free‐living form (Figure 2c). Macroalgae and cyanobacteria growing epiphytically were rare and, when recorded, contributed very little biomass.

**TABLE 2:** List of angiosperms, macroalgae, and cyanobacteria found in the study.

Group	Species
Angiospermae	*Myriophyllum spicatum*
	*Ranunculus* sp.^c^
	*Ruppia* sp.
	*Stuckenia pectinata*
	*Zannichellia palustris*
Charophyceae	*Chara* sp.
Chlorophyta	*Chaetomorpha linum* ^a^
	*Cladophora* spp.^a^
	*Cladophora fracta* ^a^
	*Cladophora glomerata* ^a^
	*Cladophora rupestris* ^a^
	*Ulothrix* sp.^a^
	*Ulva* sp.^a^
Cyanobacteria^a^	
Phaeophyceae	*Ectocarpales* sp.^b^
	*Fucus vesiculosus*
Rhodophyta	*Ceramium tenuicorne* ^b^
	*Furcellaria lumbricalis*
	*Vertebrata fucoides*

*Note:* Species in bold represent habitat‐forming macrophytes used in the calculation of species richness.

^a^ Only found as part of the drifting filamentous mat during random taxonomic checks of the whole mass, not weighted individually.

^b^ Present as epiphyte.

^c^ Rare species, found within the station, but outside the quadrats and the quantitative samples.

The filamentous mats consisted of tightly intertwined filamentous chlorophytes, dominated by *Cladophora fracta* and *Chaetomorpha linum* ; filamentous cyanobacteria were also commonly found (Table A1, Figure 2d).

### 3.2 Characteristics of the General Epifauna Community and the Herbivores

The epifaunal community sampled from all stations across the two bays comprised 6076 epifaunal individuals from 36 taxa, dominated by molluscs (68%), crustaceans (21%) and finally insects (11%) (Table A2). The most abundant organisms were Hydrobiid snails (*n* = 1723) and Cardiid bivalves (*n* = 1575), followed by the gastropod *T. fluviatilis* (*n* = 827), gammaridean amphipods (*n* = 597) and chironomid midge larvae (*n* = 596). A total of 3201 individuals belonging to the focal herbivore groups were recorded in the samples. The most abundant were the Hydrobiids (mean = 82 ± 91.3 [SD]), followed by *T. fluviatilis* (mean = 39.4 ± 49.7 [SD]), then *Gammarus* spp. (mean = 28.4 ± 42.2 [SD]) and *Idotea* spp. (mean = 2.6 ± 3.7 [SD]).

### 3.3 Influence of the Predictors on the Overall Epifaunal Community

**FIGURE 3:**
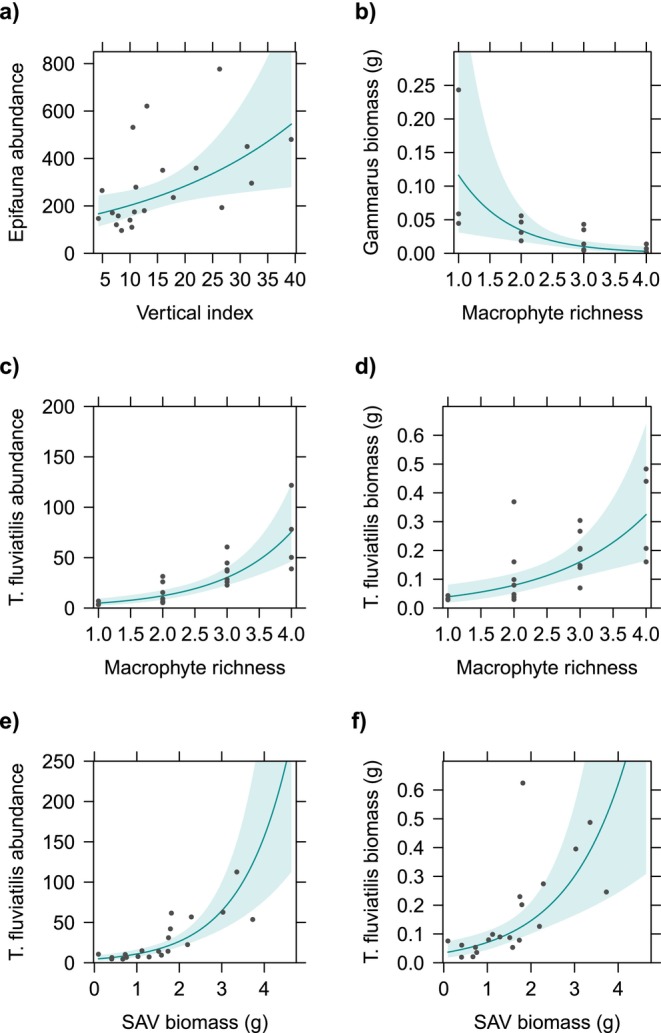
Partial regression plots displaying significant effects of benthic vegetation variables on (a) total epifauna abundance, (b) *Gammarus* and (c–f) *Theodoxus fluviatilis* . The dots represent the partial residuals and the shaded area the 95% confidence interval of the fitted values.

At the level of the overall epifaunal community, the vertical index had a significant positive effect on total epifauna abundance, with an estimated increase in the number of invertebrates by 3.44% for each unit increase in vertical index (Table 3, Figure 3a). We did not recover any significant predictors of epifaunal species richness, Shannon diversity or evenness (Table A3).

**TABLE 3:** Summary of the GLMs for (A) epifauna abundance (all species), (B) *Gammarus* biomass, (C) *Idotea* abundance, (D) Hydrobiidae (Hydrobiidae and *Potamopyrgus antipodarium*) abundance, (E) *Theodoxus fluviatilis* abundance and (F) *T. fluviatilis* biomass.

Predictor	SES	Estimate	95% CI	*p*	SES	Estimate	95% CI	*p*
	(A) Epifauna abundance				(B) *Gammarus* biomass			
Depth (m)	0.31	0.46	0.09, 0.84	0.020	−0.71	−1.1	−2.0, −0.11	0.029
Fetch (m)	−0.19	0.00	0.00, 0.00	0.2	−0.13	0.00	0.00, 0.00	0.7
Vertical index	0.34	0.03	0.01, 0.06	**0.006**	0.73	0.07	−0.01, 0.15	0.081
Filamentous mat biomass	0.30	0.26	0.07, 0.47	0.009	−0.10	−0.08	−0.56, 0.39	0.7
SAV biomass	0.12	0.10	−0.10, 0.30	0.4	−0.44	−0.38	−0.94, 0.18	0.2
Macrophyte richness	0.11	0.12	−0.13, 0.37	0.3	−1.19	−1.2	−1.9, −0.52	**< 0.001**
Location: Valleviken	0.12	0.12	0.42, 0.67	0.6	−0.41	−0.41	−1.8, 0.97	0.6
	(C) *Idotea* abundance				(D) Hydrobiidae abundance			
Depth (m)	0.04	0.06	−0.49, 0.62	0.8	0.11	0.17	−0.37, 0.74	0.5
Fetch (m)	0.14	0.00	0.00, 0.00	0.5	−0.22	0.00	0.00, 0.00	0.2
Vertical index	−0.27	−0.03	−0.07, 0.02	0.3	0.27	0.03	−0.01, 0.06	0.11
Filamentous mat biomass	−0.03	−0.02	−0.32, 0.28	0.9	0.49	0.43	0.18, 0.72	**0.001**
SAV biomass	0.39	0.33	−0.06, 0.72	0.10	−0.14	−0.12	−0.39, 0.15	0.4
Macrophyte richness	−0.23	−0.24	−0.70, 0.22	0.3	0.09	0.09	−0.24, 0.42	0.6
Location: Valleviken	1.51	1.5	0.61, 2.4	**< 0.001**	−0.44	−0.44	−1.1, 0.30	0.2
	(E) *T. fluviatilis* abundance				(F) *T. fluviatilis* biomass			
Depth (m)	0.03	0.04	−0.41, 0.50	0.9	0.11	0.17	−0.36, 0.74	0.6
Fetch (m)	0.12	0.00	0.00, 0.00	0.5	0.16	0.00	0.00, 0.00	0.5
Vertical index	0.14	0.01	−0.01, 0.04	0.4	0.10	0.01	−0.02, 0.05	0.6
Filamentous mat biomass	0.64	0.55	0.34, 0.79	**< 0.001**	0.52	0.45	0.18, 0.79	0.012
SAV biomass	1.04	0.89	0.62, 1.2	**< 0.001**	0.85	0.72	0.39, 1.1	**0.001**
Macrophyte richness	0.89	0.91	0.61, 1.2	**< 0.001**	0.69	0.71	0.33, 1.1	**0.002**
Location: Valleviken	−1.00	−1.0	−1.7, −0.33	**0.002**	−0.68	−0.68	−1.5, 0.16	0.12

*Note:* All biomasses are expressed as dry weights (g). Values in bold are significant after Bonferroni correction (significance level = 0.007).

Abbreviations: CI, confidence interval; SES, standardised effect size.

### 3.4 Influence of the Predictors on Individual Grazer Groups

*Gammarus* biomass was significantly and negatively associated with macrophyte richness, with an estimated decrease of 70.5% in biomass for every additional macrophyte species (Table 3; Figure 3b). No significant predictors of *Gammarus* abundance were recovered (Table A3). The abundance of Idotea was significantly and positively associated with location (Table 3; Figure 5a). On average, Idotea abundance was 6.3 times higher and biomass was 4.4 times larger in Valleviken compared to Lergrav.

**FIGURE 4:**
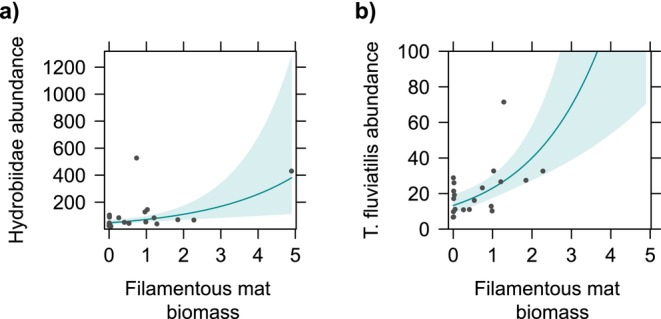
Partial regression plots displaying significant effects of filamentous mat dry biomass on the abundances of (a) Hydrobiidae and (b) *Theodoxus fluviatilis*. The group Hydrobiidae includes also *Potamopyrgus antipodarum* . The dots represent the partial residuals and the shaded area represents the 95% confidence interval of the fitted values.

**FIGURE 5:**
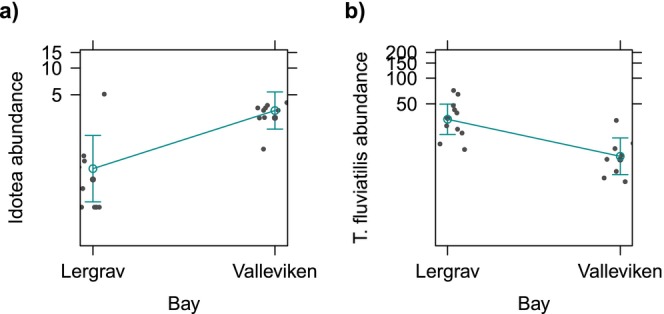
Plots of the model‐estimated effect of location (Bay) on the abundance of (a) *Idotea*, and (b) *Theodoxus fluviatilis* . The dots represent the partial residuals and the interval bounds represent the 95% confidence interval. The *y* axis has been rescaled on the fitted values for better readability.

For the gastropod grazers, hydrobiids were significantly and positively associated with the biomass of filamentous mats, with an estimated increase in abundance by 53.2% for each additional gram (dry weight) of filamentous algae (Table 3; Figure 4a). *T. fluviatilis* abundance was significantly and positively associated with SAV biomass, which had the strongest standardized effect size, followed by macrophyte richness and filamentous mat biomass (Table 3; Figures 3e,g and 4b). The number of individuals was estimated to increase by 142.2% per g dry biomass of SAV, 149.5% per each additional macrophyte species and 73.6% per g dry biomass of filamentous mat. Location showed a significant relationship with *T. fluviatilis* abundance. In contrast to isopods, *T. fluviatilis* was more abundant in Lergrav than in Valleviken (Table 3, Figure 5b). *T. fluviatilis* biomass was found to be positively and significantly associated with SAV biomass, with an estimated increase by 106.1% per g dry SAV biomass and by 102.5% per each additional vegetation species (Table 3; Figure 3f,h). No significant association was found between *T. fluviatilis* biomass and the biomass of filamentous mat (Table 3).

## 4 Discussion

The aim of this study was to investigate the importance of SAV and drifting filamentous mats in shaping epifaunal communities in shallow soft‐bottom communities on the island of Gotland, located centrally in the Baltic Sea. We also developed a rapid assessment method for quantifying vertical structure, a key aspect of habitat architecture, and tested its influence on epifaunal assemblages. SAV characteristics had positive associations with the overall epifaunal community as well as both crustaceans and gastropod grazers. Vertical structure was a strong predictor of overall epifauna abundance, supporting its use for quantifying vegetation structure. SAV biomass and macrophyte richness had positive associations with *T. fluviatilis* . In contrast, *Gammarus* biomass was negatively affected by macrophyte richness. Filamentous mats showed significant positive relationships with the gastropods only, which indicates that their influence is more specific to certain taxa. Although water depth and fetch are often key drivers in ecosystem community patterns and processes (Korpinen and Westerbom 2010; Råberg and Kautsky 2007; Wallin et al. 2011), we did not find any significant relationships between these and the macroinvertebrate community. It is possible that some of this variation was particularly captured in the predictor of ‘bay’ which had a significant effect on both *Idotea* and *T. fluviatilis* , further suggesting that other factors associated with each of the two bays have a strong role in shaping these communities that were not assessed here.

### 4.1 Importance of SAV and Other Macrophytes

This study found that SAV biomass had a significant positive effect on the abundance and biomass of *T. fluviatilis* . The positive relationship between benthic vegetation biomass and epifauna has been linked to the increased habitat complexity provided by dense vegetation and surface area available for colonisation and food, in both marine and brackish/freshwater habitats (Aleixo et al. 2022; Stoner 1980; Stoner and Lewis 1985). In this study, the pondweed *S. pectinata* contributed to 73% of the total SAV biomass (Table 3). This plant is one of the most important habitat‐forming species in soft sediments in the Baltic Sea (Kautsky 1987), and *T. fluviatilis* is typically found in association with it (Zettler et al. 2004). Moreover, although the diet of these gastropods consists mainly of macroalgae, periphyton and detritus, they are also known to consume angiosperm tissues (Jephson et al. 2008; Ziółkowska et al. 2018).

SAV vertical structure had a significant positive effect on overall epifauna abundance. Similarly to SAV biomass, vertical structure can influence epifauna by increasing habitat availability and complexity. Other than providing more space for colonisation, vertical height might also facilitate organisms that prefer different positions in the water column, for example to avoid benthic predators (Pohle et al. 1991), or parts of the vegetation that are older or younger (Hirst 2007). In our study system, percent cover of benthic vegetation was strongly dominated by *S. pectinata* , which accounted for 60% of the total benthic vegetation and 73% of the angiosperm biomass (Table 3). *S. pectinata* is characterised by elongated, narrow leaves, and it is likely that, in habitats dominated by a plant with relatively simple architecture and low leaf surface area, length provides the additional structure needed for colonisation of more abundant communities of invertebrates.

This study found contrasting effects of macrophyte species richness on the grazers, with a negative effect on *Gammarus* biomass and a positive effect on the abundance of *T. fluviatilis* . Although the importance of biodiversity in the functioning of ecosystems cannot be overestimated (Gamfeldt et al. 2015, Lefcheck et al. 2015), studies on the relationship of macrophyte species richness and invertebrates in aquatic environments have shown inconsistent, although generally positive, results (Bates and DeWreede 2007; Gustafsson and Boström 2009; Parker et al. 2001; Ramus et al. 2022). Higher vegetation richness is likely to have a significant effect on the diversity, abundance or biomass of invertebrates where it provides a wider range of micro‐habitats that can be colonised by a more diverse and abundant community (Ramus et al. 2022; Willis et al. 2005). This implies that, more than species richness by itself, what drives invertebrate communities are the morphological and physiological traits of the vegetation within the assemblage (Dencker et al. 2017; French and Moore 2018; Gustafsson and Boström 2009; Hansen et al. 2011; Moore and Duffy 2016; Parker et al. 2001; Taniguchi et al. 2003).

In this study, stations with the lowest macrophyte species richness were dominated by *S. pectinata* alone or in combination with *M. spicatum* (Table A1). Stations with progressively higher species richness maintained these as the dominant species, but further included the macroalgae *F. vesiculosus* (mostly in its free‐living form), *F. lumbricalis*, and/or the charophyta *Chara* sp. (Table A1), which provide a variety of diverse morphologies and architectures. The positive effect of species richness on *T. fluviatilis* is in line with our expectations of a positive effect of the increased diversity of habitats on epifauna. In contrast, the negative effect on *Gammarus* biomass was unexpected, given how widespread and common this species is in the Baltic Sea. Although known to feed on a variety of macrophytes, including *Fucus* and Charyophytes (Goecker and Kåll 2003; Kotta et al. 2004), *Gammarus* preferentially feeds on filamentous species (Helen et al. 2006; Kahma et al. 2021) and it is unlikely that these invertebrates are able to directly affect the distribution of macrophytes (Forslund et al. 2012). One explanation is that *Gammarus* may have a stronger affinity for certain macrophyte species. Alternatively, increased habitat diversity may have facilitated the recruitment of mesopredators that are more effective at locating and ingesting amphipods compared to isopods or gastropods, due to traits such as body shape and type of exoskeleton (Gagnon et al. 2019; Lefcheck and Duffy 2015; Moore and Duffy 2016). Field manipulations have, for instance, shown that the three‐spined stickleback ( *Gasterosteus aculeatus* ), a ubiquitous and abundant mesopredator in the Baltic Sea (Olsson et al. 2019), could reduce the biomass of amphipods by 40%–60%, while leaving gastropods unaffected (Sieben et al. 2011).

### 4.2 Importance of Filamentous Mats

Previous studies elsewhere in the Baltic Sea have found both positive and negative impacts of filamentous mats on epifauna, depending on the invertebrate species investigated and the temporal or spatial extent of the mat (Norkko and Bonsdorff 1996; Norkko et al. 2000; Salovius et al. 2005; Salovius and Kraufvelin 2004). In this study, the biomass of filamentous mats had a significant positive effect on the abundance of the two groups of gastropod grazers analyzed: Hydrobiids and *T. fluviatilis*. This is in line with other studies conducted in the near region of the Archipelago Sea (Finland), which showed that drifting filamentous mats provide habitat and food to these gastropods (Norkko et al. 2000; Salovius and Kraufvelin 2004), particularly in the period coinciding with the time of our sampling (late July, Salovius et al. 2005).

The crustacean grazers investigated here were not affected by the abundance and biomass of filamentous algal mats. This finding is in contrast with our expectations, since *Cladophora* is one of the main components of the filamentous mats in this study and, beyond providing habitat (Norkko et al. 2000), has high nutritional value (Vesakoski et al. 2008), and has also been shown to make a substantial contribution to the diet of *Gammarus* and *Idotea* (Goecker and Kåll 2003; Kahma et al. 2021). One potential explanation is that the algal mats comprised species that provide a less preferable habitat to crustacean grazers (Kraufvelin et al. 2006). Alternatively, the habitat and food value of the filamentous algae could have been reduced by the state of noticeable decay at the time of sampling. The breakdown of organic material may have also reduced oxygen levels, with the resulting hypoxia leading to emigration of the relatively more mobile (and metabolically active) crustacean grazers. In aquaria experiments, Salovius and Kraufvelin (2004) showed, for instance, that *Idotea* and juvenile *Gammarus* preferred water either free of algae or that had been in contact with fresh filamentous algae, over water that had been exposed to decaying algae. In addition, the nutritional value of the filamentous mat could have been influenced by the presence of filamentous cyanobacteria, which were commonly found in the mats. Cyanobacteria, which made a substantial contribution in most samples, have been shown to be variably utilised and processed by benthic invertebrates in the Baltic Sea (Karlson et al. 2008). Although they can represent an important food source for some meiofauna species (Nascimento et al. 2008), feeding experiments in limnic conditions showed that growth and survival of gammarids was inferior in animals fed on cyanobacteria compared to those fed on eukaryotic algae (Gergs et al. 2014).

## 5 Conclusion

This study highlights the importance of SAV in supporting diverse and abundant epifaunal invertebrate communities in the central Baltic Proper, an area historically less studied than other parts of the Baltic Sea. Macrophytes were found to promote the abundance of taxonomically and functionally diverse epifaunal communities, whereas filamentous mats were found to be important for gastropods only. As many fishes and other mesopredators display species‐specific preferential predation on different invertebrates (Gagnon et al. 2019; Lappalainen et al. 2001; Lefcheck and Duffy 2015), changes in epifaunal communities can lead to changes in energy and nutrient uptake at higher trophic levels, with potential impacts on coastal food webs.

As the Baltic Sea continues to change, monitoring the invertebrate communities as well as their SAV and algal resources will be crucial to understanding ecosystem functionality. The vertical structure index presented in this study offers a valuable tool for monitoring this important aspect of vegetation structure and how it translates to the epifaunal community.

## Author Contributions

**Chiara D'Agata:** conceptualization (lead), data curation (lead), formal analysis (lead), investigation (lead), methodology (supporting), visualization (lead), writing – original draft (lead), writing – review and editing (equal). **Thomas A. B. Staveley:** conceptualization (supporting), formal analysis (supporting), methodology (supporting), supervision (supporting), writing – original draft (supporting), writing – review and editing (equal). **Johan S. Eklöf:** conceptualization (supporting), formal analysis (supporting), methodology (supporting), writing – original draft (supporting), writing – review and editing (equal). **Jonathan S. Lefcheck:** formal analysis (supporting), methodology (supporting), writing – original draft (supporting), writing – review and editing (equal). **Gunilla Rosenqvist:** investigation (supporting), resources (supporting), writing – original draft (supporting), writing – review and editing (supporting). **Lina Mtwana Nordlund:** conceptualization (supporting), funding acquisition (lead), investigation (supporting), supervision (supporting), writing – review and editing (supporting).

## Conflicts of Interest

The authors declare no conflicts of interest.

## Data Availability

Data and R scripts are available from Figshare at https://doi.org/10.6084/m9.figshare.28053197

## Appendix A

**TABLE A1:** Dry weight (g^−sample^), average percent cover (±SD), and species richness of macrophytes at each sampling station in Valleviken and Lergrav.

Bay	Station	Measure	*Char*	*Fuc ves*	*Fur lum*	*Myr spi*	*Ranu*	*Rupp*	*Stu pec*	*Zan pal*	Species richness
Valleviken	V1	gDW/sample							1.12		3
		% cover (±SD)		20 (±20)		0.33 (±0.58)			11.67 (±16.07)		
	V2	gDW/sample			0.01	0.03			3.00		3
		% cover (±SD)							25 (±0)		
	V3	gDW/sample							4.64		1
		% cover (±SD)							31.67 (±7.64)		
	V4	gDW/sample						1.24		0.04	2
		% cover (±SD)						28.33 (±28.43)		20.0 (±30.41)	
	V6	gDW/sample				1.80			0.02		2
		% cover (±SD)				13.33 (±11.55)			15.00 (±8.66)		
	V7	gDW/sample							1.03		1
		% cover (±SD)							56.67 (±30.55)		
	V8	gDW/sample	0.08						0.02	0.39	3
		% cover (±SD)	1.67 (±2.89)						51.67 (±23.63)		
	V9	gDW/sample				0.21			1.53		4
		% cover (±SD)		1.67 (±2.89)	0.33 (±0.58)	3.33 (±2.89)			1 (±0)		
	V10	gDW/sample				0.28			0.13		4
		% cover (±SD)		3.33 (±5.77)		6.67 (±5.77)	1.67 (±2.89)		2.33 (±2.31)		
	V11	gDW/sample							0.67		2
		% cover (±SD)				1.67 (±2.89)			8.33 (±2.89)		
Lergrav	L1	gDW/sample						1.51			3
		% cover (±SD)		0.33 (±0.58)				13.33 (±12.58)	13.67 (±18.58)		
	L2	gDW/sample				0.77			0.81		4
		% cover (±SD)	1.67 (±2.89)	0.33 (±0.58)		1.67 (±2.89)			43.33 (±28.43)		
	L3	gDW/sample							3.35		2
		% cover (±SD)				6.67 (±7.64)			21.67 (±5.77)		
	L4	gDW/sample	0.04						0.10		2
		% cover (±SD)	3.33 (±2.89)						6.67 (±11.55)		
	L5	gDW/sample							1.75		3
		% cover (±SD)		0.33 (±0.58)		1.67 (±2.89)			2 (±8.66)		
	L6	gDW/sample				0.01			0.75		2
		% cover (±SD)							18.33 (±5.77)		
	L7	gDW/sample							3.73		2
		% cover (±SD)				0.33 (±0.58)			48.33 (±22.55)		
	L8	gDW/sample		2.92		0.85			0.94		3
		% cover (±SD)		8.33 (±5.77)		30 (±21.79)			36.67 (±11.55)		
	L9	gDW/sample							2.19		1
		% cover (±SD)							5 (±1)		
	L10	gDW/sample				0.29		0.17	0.27		3
		% cover (±SD)				13.33 (±7.64)			15.00 (±8.66)		
	L11	gDW/sample		4.25		1.72		0.40	0.17		4
		% cover (±SD)		45 (±32.79)		21.67 (±12.58)			25.00 (±22.91)		

Abbreviations: *Char*, *Chara* spp.; *Fuc ves*, *Fucus vesiculosus* ; *Fur lum*, *Furcellaria lumbricalis*; *Myr spi*, *Myriophyllum spicatum* ; *Ranu*, *Ranunculus* sp.; *Stu pec*, *Stuckenia pectinata* ; *Zan pal*, *Zannichellia palustris* .

**TABLE A2:** Taxa of the epifauna community, including their abundance and frequency of stations in Valleviken, Lergrav, and all stations, along with total dry biomass (gDW) and abundance.

Group	Species	Valleviken (*n* = 10)	Lergrav (*n* = 11)	Total (*n* = 21)	Total biomass (gDW)	Total abundance
Bryozoa	*Einhornia crustulenta*	2 (20%)	0	2 (10%)	0.0020	2
Crustacea	*Amphibalanus improvisus*	2 (20%)	0	2 (10%)	0.0020	2
	*Asellus aquaticus*	1 (10%)	0	1 (5%)	0.0005	1
	*Corophium volutator*	0	3 (27%)	3 (14%)	0.0047	5
	*Crangon crangon*	1 (10%)	0	1 (5%)	0.0097	1
	*Gammarus locusta*	2 (20%)	0	2 (10%)	0.0059	2
	*Gammarus salinus*	4 (40%)	1 (9%)	5 (24%)	0.0109	7
	*Gammarus tigrinus*	5 (50%)	3 (27%)	8 (38%)	0.0645	24
	*Gammarus* spp.	8 (80%)	10 (91%)	18 (86%)	0.5173	564
	**Total *Gammarus***				**0.5986**	**597**
	*Heterotanais oerstedii*	5 (50%)	2 (18%)	7 (33%)	0.0178	321
	*Idotea balthica*	3 (30%)	1 (9%)	4 (19%)	0.0194	9
	*Idotea chelipes*	7 (70%)	4 (36%)	11 (52%)	0.0829	24
	*Idotea granulosa*	3 (30%)	0	3 (14%)	0.0139	5
	*Idotea* spp.	3 (30%)	1 (9%)	4 (19%)	0.0123	16
	**Total *Idotea***				**0.1285**	**54**
	*Jaera albifrons*	1 (10%)	0	1 (5%)	0.0010	1
	*Lekanesphaera rugicauda*	8 (80%)	10 (91%)	18 (86%)	0.3390	274
Hydrozoa	*Cordylophora*	1 (10%)	1 (9%)	2 (10%)	0.0020	2
Insecta	Diptera	2 (20%)	0	2 (10%)	0.0017	3
	*Cataclysta*	1 (10%)	0	1 (5%)	0.0032	4
	Ceratopogonidae	1 (10%)	0	1 (5%)	0.0010	1
	Chironomidae	9 (90%)	8 (73%)	17 (81%)	0.0509	596
	Coleoptera	1 (10%)	1 (9%)	2 (10%)	0.0037	2
	Corixidae	0	3 (27%)	3 (14%)	0.0471	15
	*Donacia*	1 (10%)	0	1 (5%)	0.0043	2
	Hydroptilidae	4 (40%)	0	4 (19%)	0.0044	5
	Lepidoptera	3 (30%)	5 (45%)	8 (38%)	0.0074	16
	Trichoptera	1 (10%)	4 (36%)	5 (24%)	0.0059	11
	Zygoptera	1 (10%)	5 (45%)	6 (29%)	0.0103	9
Mollusca	Cardiidae	10 (100%)	11 (100%)	21 (100%)	3.1141	1575
	Hydrobiidae	10 (100%)	11 (100%)	21 (100%)	1.9828	1668
	*Potamopyrgus antipodarum*	7 (70%)	7 (64%)	14 (67%)	0.1155	55
	**Total Hydrobiidae and *P. antipodarum***				**2.0983**	**1723**
	*Limapontia capitata*	1 (10%)	0	1 (5%)	0.0004	1
	*Mya arenaria*	1 (10%)	0	1 (5%)	0.0637	1
	*Mytilus edulis*	1 (10%)	0	1 (5%)	0.0019	1
	*Ampullaceana balthica*	3 (30%)	2 (18%)	5 (24%)	0.1177	24
	***Theodoxus fluviatilis***	**10 (10%)**	**11 (100)**	**21 (100%)**	**3.8590**	**827**

*Note:* Focus grazers are in bold.

**TABLE A3:** Summary of the GLMs for the models of total epifauna diversity (richness, Hill numbers and Pielou's evenness), *Gammarus* abundance, biomass of *Idotea* and biomass of Hydrobiidae.

Predictors	Estimate	95% CI	*p*	Estimate	95% CI	*p*	Estimate	95% CI	*p*
	Epifauna richness			Epifauna Hills			Epifauna Pielou's evenness		
Depth (m)	−0.11	−0.36, 0.14	0.4	−0.14	−0.41, 0.08	0.2	−0.14	−0.44, 0.16	0.4
Fetch (m)	0.00	0.00, 0.00	0.6	0.00	0.00, 0.00	0.2	0.00	0.00, 0.00	0.4
Vertical index	−0.01	−0.02, 0.01	0.4	0.00	−0.02, 0.01	0.9	0.01	−0.01, 0.02	0.6
Filamentous mat biomass	0.09	−0.02, 0.20	0.10	0.01	−0.12, 0.10	0.9	−0.08	−0.23, 0.07	0.3
SAV biomass	0.07	−0.07, 0.21	0.3	0.01	−0.12, 0.15	0.8	−0.04	−0.21, 0.13	0.7
Macrophyte richness	−0.11	−0.27, 0.04	0.15	−0.04	−0.19, 0.10	0.5	0.04	−0.15, 0.22	0.7
Bay									
Lergrav	—	—		—	—				
Valleviken	0.17	−0.15, 0.49	0.3	−0.06	−0.40, 0.25	0.7			
BayValleviken							−0.21	−0.60, 0.19	0.3
	*Gammarus* abundance			*Idotea* biomass			Hydrobiidae biomass		
Depth (m)	−0.08	−1.3, 1.1	> 0.9	0.24	−0.61, 1.2	0.6	0.03	−0.56, 0.67	> 0.9
Fetch (m)	0.00	0.00, 0.00	0.5	0.00	0.00, 0.00	0.3	0.00	0.00, 0.00	0.6
Vertical index	0.05	−0.04, 0.14	0.2	−0.04	−0.11, 0.05	0.2	0.00	−0.04, 0.05	0.9
Filamentous mat biomass	0.02	−0.58, 0.63	> 0.9	−0.16	−0.58, 0.40	0.5	0.36	0.07, 0.72	0.044
SAV biomass	−0.50	−1.2, 0.15	0.13	0.01	−0.53, 0.57	> 0.9	−0.06	−0.37, 0.26	0.7
Macrophyte richness	−0.91	−1.7, −0.10	0.027	−0.27	−0.76, 0.23	0.3	0.07	−0.32, 0.47	0.7
Bay									
Lergrav	−0.22	−1.8, 1.4	0.8						
Valleviken				—	—		—	—	
BayValleviken				1.1	0.01, 2.2	0.086	−0.10	−0.93, 0.78	0.8

*Note:* All biomasses are expressed as dry weights (g).

Abbreviation: CI, confidence interval.
